# A Randomized Controlled Trial to Examine the Effect of 2-Year Vitamin B12 and Folic Acid Supplementation on Physical Performance, Strength, and Falling: Additional Findings from the B-PROOF Study

**DOI:** 10.1007/s00223-015-0059-5

**Published:** 2015-09-28

**Authors:** Karin M. A. Swart, Annelies C. Ham, Janneke P. van Wijngaarden, Anke W. Enneman, Suzanne C. van Dijk, Evelien Sohl, Elske M. Brouwer-Brolsma, Nikita L. van der Zwaluw, M. Carola Zillikens, Rosalie A. M. Dhonukshe-Rutten, Nathalie van der Velde, Johannes Brug, André G. Uitterlinden, Lisette C. P. G. M. de Groot, Paul Lips, Natasja M. van Schoor

**Affiliations:** Department of Epidemiology and Biostatistics and the EMGO Institute for Health and Care Research, VU University Medical Center, Van der Boechorststraat 7, 1081 BT Amsterdam, The Netherlands; Department of Internal Medicine, Erasmus MC, P.O. Box 2040, 3000 CA Rotterdam, The Netherlands; Department of Human Nutrition, Wageningen University, P.O. Box 8129, 6700 EV Wageningen, The Netherlands; Department of Internal Medicine, Endocrine Section and the EMGO Institute for Health and Care Research, VU University Medical Center, P.O. Box 7057, 1007 MB Amsterdam, The Netherlands

**Keywords:** Homocysteine, Aging, Falling, Physical function, Vitamin B12, Folic acid

## Abstract

Elevated homocysteine concentrations are associated with a decline in physical function in elderly persons. Homocysteine-lowering therapy may slow down this decline. This study aimed to examine the effect of a 2-year intervention of vitamin B12 and folic acid supplementation on physical performance, handgrip strength, and risk of falling in elderly subjects in a double-blind, randomized placebo-controlled trial. Participants aged ≥65 years with elevated plasma homocysteine concentrations [12–50 µmol/L (*n* = 2919)] were randomly assigned to daily supplementation of 500 µg vitamin B12, 400 µg folic acid, and 600 IU vitamin D3, or to placebo with 600 IU vitamin D3. Physical performance (range 0–12) and handgrip strength (kg) were measured at baseline and after 2 years. Falls were reported prospectively on a research calendar. Intention-to-treat (primary) and per-protocol (secondary) analyses were performed. Physical performance level and handgrip strength significantly decreased during the follow-up period, but this decline did not differ between groups. Moreover, time to first fall was not significantly different (HR: 1.0, 95 % CI 0.9–1.2). Secondary analyses on a per-protocol base identified an interaction effect with age on physical performance. In addition, the treatment was associated with higher follow-up scores on the walking test (cumulative OR: 1.3, 95 % CI 1.1–1.5). Two-year supplementation of vitamin B12 and folic acid was neither effective in reducing the age-related decline in physical performance and handgrip strength, nor in the prevention of falling in elderly persons. Despite the overall null-effect, the results provide indications for a positive effect of the intervention on gait, as well as on physical performance among compliant persons >80 years. These effects should be further tested in future studies.

## Introduction

Poor physical function in the elderly is associated with adverse health outcomes, including falls and fractures, a reduced quality of life, nursing home admission and mortality [1–5]. Circulating homocysteine (Hcy) concentrations increase with age, and elevated concentrations occur in up to 50 % of persons over the age of 60 [6]. Elevated Hcy levels have been identified as independent risk factor for fractures [7, 8]. In addition, Hcy has been suggested to have effects on multiple systems, including vascular and neuromuscular systems, and these systems may affect mobility and gait. Reduction in muscle strength, physical performance, and falling may be an intermediate pathway by which high Hcy induces an increased fracture risk in older persons. In the past decade, the association between Hcy and physical function has been examined in several cross-sectional and longitudinal observational studies. These studies have consistently shown that elevated Hcy concentrations are associated with accelerated decline in mobility and muscle strength [9–15]. Hcy-induced neurological deterioration or adverse muscle characteristics are thought to account for the observed associations [16, 17].

Elevated Hcy concentrations can effectively be reduced with vitamin B12 and/or folic acid supplementation [18]. If the relation between Hcy and physical function is causal, supplementation can be expected to prevent or slow down decline in physical function in the elderly, and this should be tested in randomized controlled trials. One such intervention study investigated the effect of randomly assigned B-vitamin supplementation versus placebo on movement performance in a population of elderly [19], and did not demonstrate a difference. However, participants were only followed for 4 months, which might be too short for physical decline to occur. A second intervention study reported no difference in fall rate after 2 years of vitamin B12 and folic acid supplementation versus placebo in a highly selective population of stroke survivors [20].

The aim of the current study was to investigate the effect of a 2-year intervention of daily vitamin B12 and folic acid supplementation on changes in physical performance, handgrip strength, and the risk of falling in a mildly hyperhomocysteinemic elderly population.

## Subjects and Methods

### Design and Study Sample

The B-vitamins in the prevention of osteoporotic fractures (B-PROOF) study is a double-blind, randomized, placebo-controlled trial. The main outcome of the trial was the incidence of osteoporotic fractures; physical performance, handgrip strength, and falls were pre-specified secondary outcomes. The study design of the B-PROOF study has been published elsewhere [21]. In short, the intervention comprised daily oral supplementation of 500 µg vitamin B12, 400 µg folic acid, and 600 IU vitamin D for a period of 2 years. The placebo tablet contained 600 IU vitamin D only. Vitamin D was added to ensure normal vitamin D concentrations. The tablets were indistinguishable with respect to smell, taste, and appearance. The random allocation sequence was computer-generated. Randomization was carried out by an independent research dietician to a one-by-one allocation ratio in blocks of 24, after stratification for sex, age (65–80, ≥80 years), study location, and baseline Hcy concentration (12–18 µmol, ≥18 µmol). Both the participants and the research team, including examiners and data-analyzers, were blinded for the treatment allocation. Persons were included if they were aged ≥65 years, and had plasma Hcy concentration of 12–50 µmol/L. Persons were excluded if they had a history of cancer in the last 5 years, except non-melanoma skin cancer, if they were bedridden or wheelchair bound, had a serum creatinine concentration >150 µmol/L, or if they currently or recently (<4 months) used supplements with very high doses of vitamin B12 (intramuscular injections) or folic acid (300 µg/day). A total of 2919 participants were included from October 2008 to March 2011. Subsequently, follow-up was completed in March 2013. Measurements were performed at baseline and after 2 years by trained examiners. Furthermore, the participants recorded falls and fractures prospectively on a research calendar. Persons who dropped-out during follow-up were asked if they agreed to continue to complete the calendar and to participate at the 2-year follow-up measurements.

The B-PROOF study was carried out by a consortium of researchers from Wageningen University (WU), VU University Medical Center (VUmc), and Erasmus MC, the Netherlands. The B-PROOF study was approved by the Medical Ethics Committee of WU, and the Medical Ethics Committees of VUmc and Erasmus MC gave additional approval for local feasibility. All participants gave written informed consent. B-RPOOF is registered with the Netherlands Trial Register (NTR1333), and the ClinicalTrials.gov (NCT 00696514).

### Outcomes

Physical performance was assessed with three function tests. During the walking test, the time needed to walk three meters back and forth as quickly as possible was measured. The chair stands contained the measurement of the time needed to stand up from and sit down on a chair for five successive times, without using hands. During the tandem stand, the ability to stand with the feet right in front of each other for 10 s with eyes open was measured. Scores on the walking test (0–4) and the chair stands (0–4) were based on timed quartiles of the study sample [14]. The tandem stand was categorized as follows: unable or able to hold position less than 4 s (score 0), able to hold position for 4–9 s (score 2), able to hold position for at least 10 s (score 4). Total physical performance score was calculated by summing up the scores of the three individual components, and consequently ranged from zero (low physical performance) to twelve (high physical performance). These physical performance tests have been used in the Longitudinal Aging Study Amsterdam and a lower score have been associated with adverse outcomes, such as functional decline and institutionalization [22], an increased fracture risk [4], and depression [23] in older persons.

Handgrip strength (kg) was measured with a hand held dynamometer (Takei TKK 5401, Takei Scientific Instruments CO. Ltd., Tokyo, Japan). Two maximum handgrip strength trials were performed with each hand. Handgrip strength was calculated as the mean of the highest scores of both hands.

Falling was assessed prospectively during the study period. Participants reported falls weekly on the research calendar. Calendar pages were returned to the study centers every 3 months. Participants were contacted if calendars were incomplete or unclear. Drop-outs with no further calendar information after drop-out were assigned as lost-to-follow-up. Time to first and time to second fall were regarded as outcomes as well as the number of falls per individual.

### Compliance

Participants returned their remaining supplement tablets bi-annually. Compliance was assessed by count of the returned tablets. Two different definitions of compliance were used. First, concerning physical performance and handgrip strength, compliance was defined as taking at least 80 % of the tablets in the 2-year period between baseline and follow-up. Second, concerning time to falling, 80 % was used as cutoff for compliance for the time the participant actually participated (i.e., time to study completion, or time to drop-out).

### Baseline Characteristics

Plasma Hcy, serum holotranscobalamin, methylmalonic acid, vitamin B12, and folate were determined. Details of the determination were described previously [21]. To assure the quality of the Hcy measurement during the inclusion period of the study, Hcy was measured in series, and control samples were analyzed during all series. Smoking habits (former, current, no smoker), alcohol use (light, moderate, excessive), physical activity, education level (low, intermediate, high), B12 and/or folic acid supplement use, and retrospective falling were assessed with questionnaires. Standing height was measured using a stadiometer, and weight using a calibrated scale (Seca, Deventer, the Netherlands). Cardiovascular disease was self-reported and was defined as the presence of arrhythmia, angina pectoris, myocardial infarction, heart failure/valve dysfunction, arterial septum defect, pericarditis, aneurysm, or pulmonal hypertension. Global cognitive functioning was measured with the Mini Mental State Examination.

### Statistical Analyses

For physical performance, a decline of 0.6 points is expected in 2 years in the placebo group (adapted from [15]), and we expect this decline to be prevented in the intervention group. Based on this expected difference of 0.6 between the treatment groups, a standard deviation of 3.2 and a power of 80 % to detect this difference, 448 participants per group would be needed. Similarly, a decline in handgrip strength of 2.3 kg is expected in 2 years in the placebo group (adapted from [13]), and we expect this decline to be prevented in the intervention group. With a power of 80 %, and standard deviation of 10.0, 298 participants in both groups are needed.

Analyses were performed using IBM SPSS Statistics 20 (IBM, Armonk, New York, United States). Comparisons of baseline characteristics between treatment groups have been made previously [24]. Additional baseline comparisons between the intervention and placebo group were made using a *t* test for physical performance and handgrip strength, using a *χ*^2^ test for percentage of retrospective fallers and the presence of cardiovascular disease, and using a Mann–Whitney *U* test for cognitive functioning.

The primary analyses were performed according to the intention-to-treat (ITT) principle, in which all participants were included. With respect to physical performance and handgrip strength, linear mixed model analyses were performed to assess the effect of the treatment. An important feature of this model, necessary for longitudinal analyses, is that the dependency of repeated observations within subjects is taken into account. Moreover, all subjects with at least one observation are included, regardless of missing values. Thus, subjects with baseline data who were lost to follow-up were also included in the analyses. The treatment effect is the mean difference from baseline to follow-up in the treatment group compared with the mean difference from baseline to follow-up in the placebo group, as defined by the treatment-by-time interaction.

With respect to falling, differences in time to first and time to second fall between treatment groups were tested with the crude log-rank test. Moreover, Cox proportional hazards analyses were performed to assess the HR of falling. Person-time was the time to first/second fall, time to lost-to-follow-up, or time to study completion, whichever came first. The assumptions of proportional hazards were assessed by visual judgment of the log-minus-log survival plots, and were not violated. To compare the number of falls per participant across treatment groups while allowing for multiple events, we used the negative binomial model.

Crude analyses were performed, and analyses adjusted for age, sex, study center, baseline Hcy, and variables that differed between groups at baseline, i.e., baseline holotranscobalamin. The analyses of the number of falls were additionally adjusted for participation time, i.e., time to lost-to-follow-up, or time to study completion. Interaction of age (below and above 80 years), sex, and baseline Hcy concentration (below and above 18 µmol/L) with the treatment effect were studied, and in addition the interaction with baseline physical performance (below and above two points), baseline handgrip strength (below and above 16 kg), or B12 and/or folic acid supplement use was tested in post hoc analyses. A *p* value of ≤0.10 for interaction was considered as a justification for stratified analyses.

In secondary analyses, the association between change in Hcy (instead of treatment allocation) and the outcomes was studied using regression analyses. In addition, ordinal logistic regression was used to examine the association between the treatment and the individual performance tests (walking test, chair stands and tandem stand). Assumptions of ordinal logistic regression were tested by the test of the parallel lines and were fulfilled if the two lowest categories were combined.

The main analyses were repeated according to the per-protocol (PP) principle, in which only compliant participants were included. Significance level of the effect was set at *p* < 0.05.

## Results

**Fig. 1:**
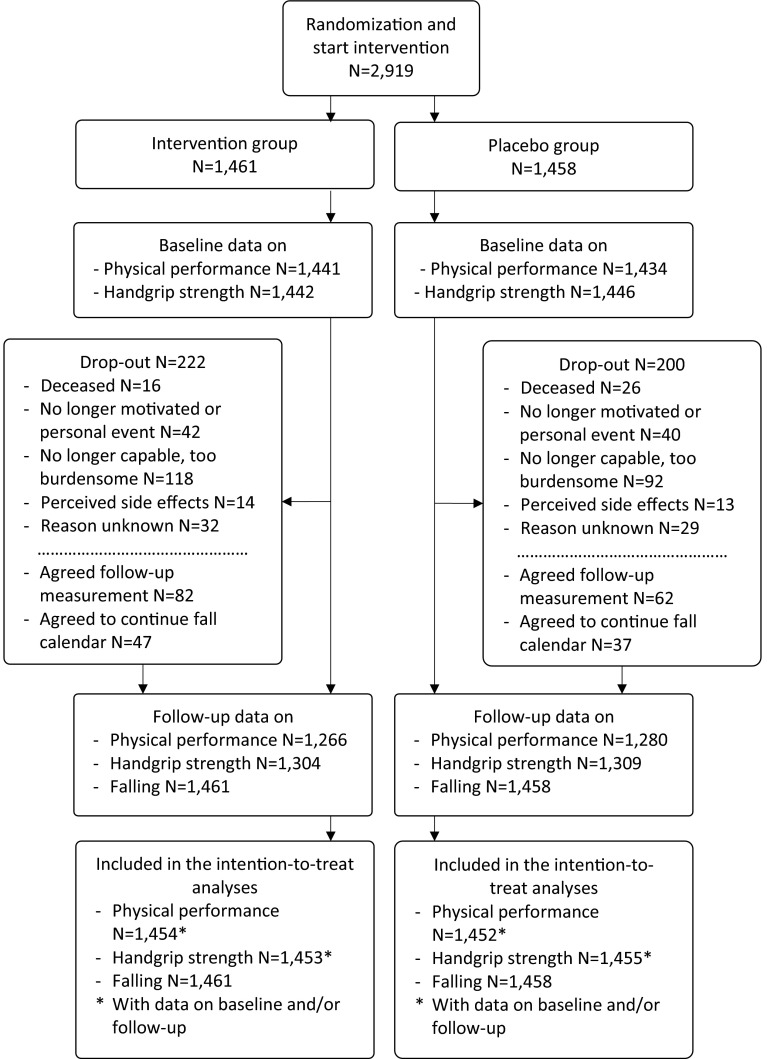
Flow chart of the B-PROOF study sample

Of the 2919 included participants, 1461 were assigned to the intervention group, and 1458 to the placebo group. The drop-out rate was 14.5 %. Among the drop-outs, 144 participants (34 %) agreed to complete the follow-up measurements, and 84 drop-outs (20 %) still completed the research calendar (Fig. 1). Baseline comparisons between the intervention and placebo group are presented in Table 1. Data on 2-year changes in Hcy, folate, holotranscobalamin, vitamin B12, and methylmalonic acid have been published previously [25].

**Table 1:** Baseline characteristics of the 2919 participants of the B-PROOF study according to treatment group

	Placebo group *N* = 1458	Intervention group *N* = 1461	*p* value
Age (years)^a^	74.0 ± 6.6	74.2 ± 6.4	0.60
Sex (% women)^b^	50.4	49.7	0.71
Homocysteine (µmol/L)^c^	14.3 (13.0–16.5)	14.5 (13.0–16.7)	0.46
Holotranscobalamin (pmol/L)^c^	63.0 (45.0–84.0)	65.0 (48.0–86.0)	0.03*
Methylmalonic acid (µmol/L)^c^	0.23 (0.18–0.31)	0.22 (0.18–0.30)	0.25
Vitamin B12 (pmol/L)^c^	266 (204–343)	267 (213–341)	0.27
Folate (nmol/L)^c^	18.9 (14.8–24.5)	18.8 (14.9–24.7)	0.50
Creatinine (µmol/L)^a^	84.1 ± 18.0	83.9 ± 18.6	0.73
Height (cm)^a^	169.2 ± 9.3	169.4 ± 9.4	0.63
Weight (kg)^a^	77.8 ± 13.3	77.9 ± 13.3	0.99
Current smoker (%)^b^	9.7	9.5	0.97
Alcohol use^b^			0.46
Light (%)	66.8	68.0	
Moderate (%)	29.0	28.5	
Excessive (%)	4.2	3.5	
Physical activity (min/day)^c^	131 (86–193)	126 (81–190)	0.17
Education^b^			0.79
Low (%)	53.6	52.4	
Intermediate (%)	21.1	21.1	
High (%)	25.4	26.5	
B12 and/or folic acid supplement use (% yes)^b^	15.8	15.3	0.76
Vitamin D supplement use (% yes)^b^	19.7	18.3	0.64
Retrospective falls (% yes)^b^	32.6	32.5	0.96
Physical performance (0–12)^c^	8.1 ± 3.2	8.0 ± 3.1	0.37
Walking test	3 (2–4)	3 (2–4)	0.08
Chair stands	3 (2–4)	3 (2–4)	0.39
Tandem stand	4 (0–4)	4 (0–4)	0.43
Handgrip strength (kg)^a^	31.0 ± 10.6	30.8 ± 10.6	0.71
Study center^b^			0.91
WU (%)	29.2	29.6	
VUmc (%)	26.4	26.8	
Erasmus MC (%)	44.4	43.6	
Cardiovascular disease (% yes)	25.0	24.1	0.46
MMSE score	28 (27–29)	28 (27–29)	0.10

*VUmc* VU University Medical Center, *WU* Wageningen University, *MMSE* mini-mental state examination

* *p* < 0.05

^a^Presented as mean ± SD, difference tested using *t* test

^b^Presented as percentages, differences tested using *χ* ^2^ test

^c^Presented as median (IQR), differences tested using Mann–Whitney *U* test

A significant 2-year decline in physical performance score was observed in both the intervention (*p* < 0.01) and placebo group (*p* < 0.01). Linear mixed models did not show a significant treatment effect, indicating that the decline did not differ between treatment groups (treatment effect: 0.1; 95 % CI −0.1–0.3) (Table 2). No interactions with age, sex, and baseline Hcy concentration were observed. In addition, the post hoc interaction with baseline physical performance or B12 and/or folic acid supplement use was not significant.

**Table 2:** The effect of the intervention on physical performance and handgrip strength, as derived from linear mixed models

	Intervention group			Placebo group			Treatment effect^a^ (95 % CI)	SE	*p* value
	Baseline estimated mean	Follow-up estimated mean	2-Year change	Baseline estimated mean	Follow-up estimated mean	2-year change			
Physical performance score									
Intention-to-treat	8.0	7.6	−0.4	8.1	7.6	−0.5	0.1 (−0.1 to 0.3)	0.10	0.36
Per-protocol	8.3	7.9	−0.4	8.3	7.9	−0.5	0.1 (−0.1 to 0.3)	0.10	0.33
Handgrip strength (kg)									
Intention-to-treat	30.9	29.6	−1.3	30.9	29.5	−1.4	0.1 (−0.2 to 0.4)	0.14	0.48
Per-protocol	31.7	30.5	−1.3	31.7	30.2	−1.5	0.2 (−0.1 to 0.5)	0.15	0.15

*CI* confidence interval, *SE* standard error

^a^The treatment effect is the mean difference from baseline to follow-up in the intervention group compared with the mean difference from baseline to follow-up in the placebo group, as determined by the treatment-by-time estimate

**Fig. 2:**
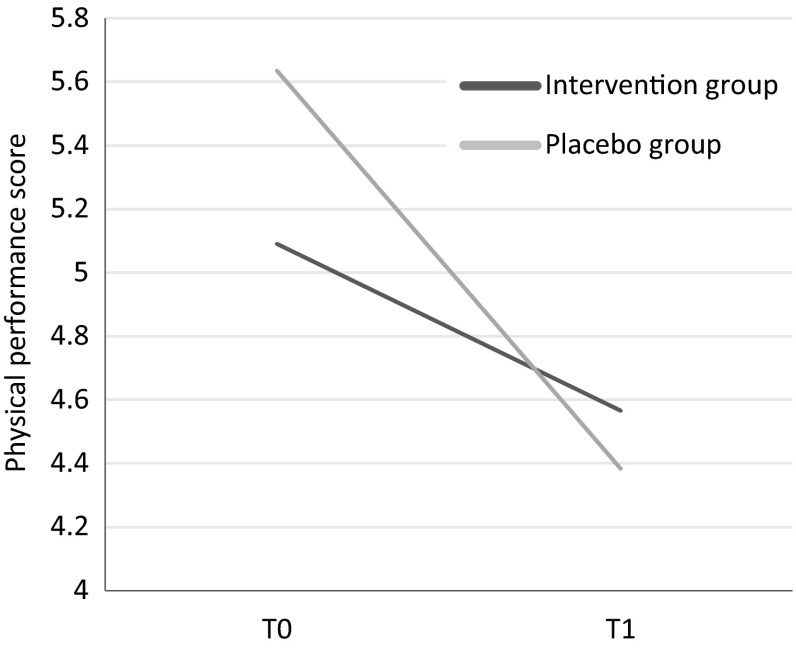
Two-year decline in physical performance scores according to treatment group among participants >80 years, as derived from linear mixed models (per-protocol analysis)

The PP analyses, with 84 % of the participants included, showed similar results (Table 2). In the PP analyses, evidence for interaction with age was observed (*p* = 0.10). Stratified analyses showed that in persons ≤80 years (*n* = 2107) the decline in physical performance score was not significantly different between treatment groups (treatment effect: 0.0; 95 % CI −0.2–0.3). In persons >80 years (*n* = 340), a treatment effect of 0.6 was observed (95 % CI 0.0–1.1), which was close to statistical significance (Fig. 2). Results were similar after adjustments for age, sex, study center, baseline Hcy, and baseline holotranscobalamin (data not shown).

Secondary analyses revealed that the 2-year change in Hcy (median: −2.4 µmol/L, IQR: −4.7 to 0.1 µmol/L) was significantly associated with follow-up physical performance scores (B: −0.03; 95 % CI −0.05 to −0.01). When testing the association between the treatment and the individual physical performance tests, the cumulative OR for the walking test was 1.3 (95 % CI 1.1–1.5), indicating that persons in the placebo group were 1.3 times more likely to score 1 point lower on the walking test, as compared with persons in the treatment group. For the chair stands and the tandem stand the cumulative ORs were 1.0 (95 % CI 0.8–1.1) and 0.9 (95 % CI 0.7–1.1), respectively.

Regarding handgrip strength, a significant 2-year decline in both treatment groups was observed (*p* < 0.01 for both groups). The decline was not significantly different between groups (treatment effect: 0.1; 95 % CI −0.2–0.4) (Table 2). No interaction effects were observed. PP results were similar (Table 2), and also adjustments for confounders did not change the findings (data not shown). Also the 2-year change in Hcy was not associated with follow-up handgrip strength (B: −0.01; 95 % CI −0.05–0.02).

**Fig. 3:**
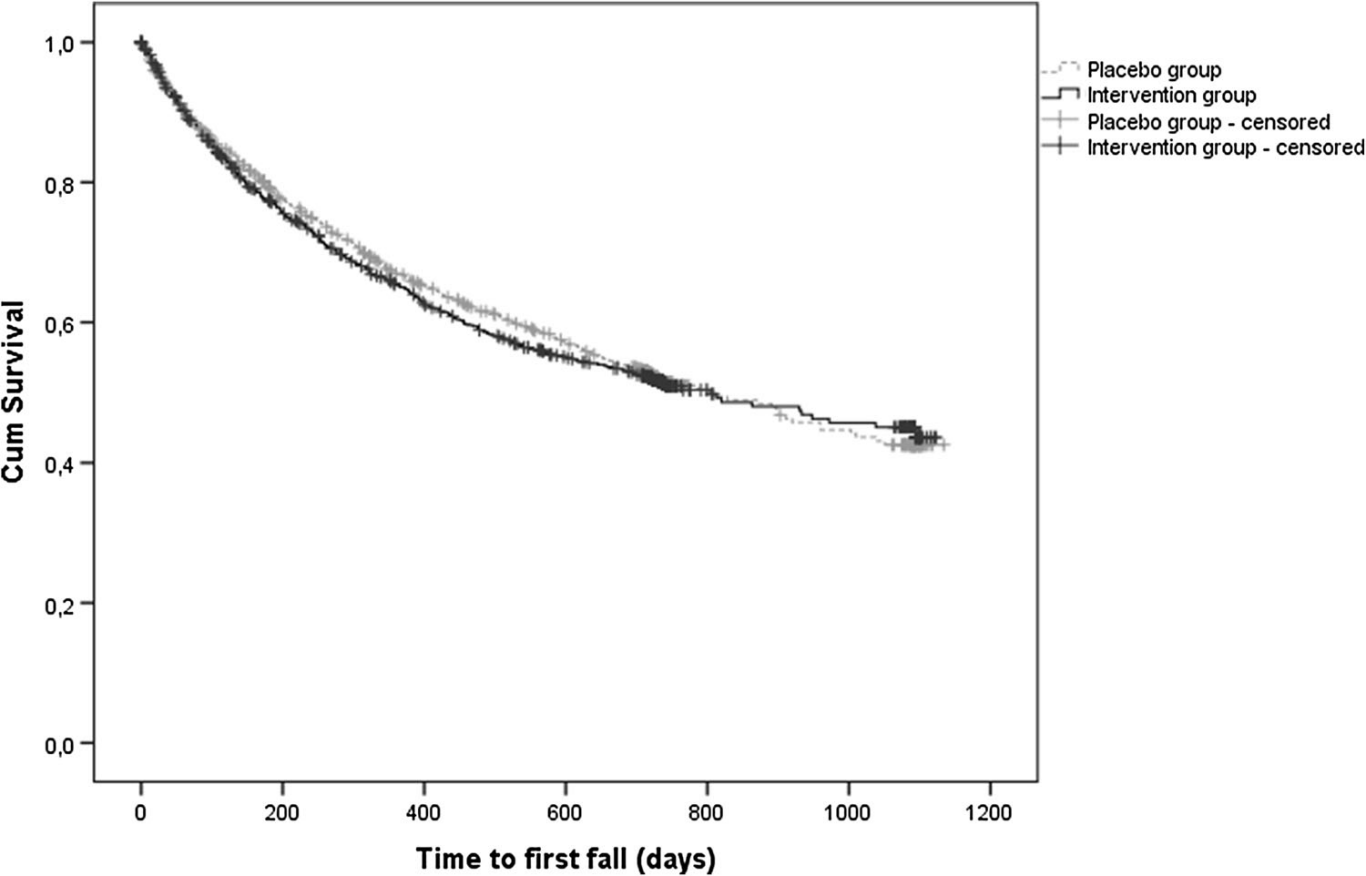
Kaplan-Meier plot of the first fall according to treatment group (intention-to-treat).

Total follow-up time for falling was 2914.7 person-years in the intervention group and 2940.7 person-years in the placebo group. In the intervention group, 1747 falls occurred in 683 fallers (fall rate: 59.9/100 person-years) versus 1663 falls in 681 fallers (fall rate: 56.6/100 person-years) in the placebo group. Both time to the first fall and time to the second fall were not significantly different between the intervention and placebo group (log rank *p* = 0.63 and *p* = 0.23, respectively) (Fig. 3 for time to first fall). Also, Cox proportional hazards models did not show statistically significant differences between treatment groups with respect to time to first fall (HR: 1.0; 95 % CI 0.9–1.2) and time to second fall (HR: 1.1; 95 % CI 0.9–1.3). For time to first fall, a significant interaction was observed with Hcy concentration (*p* = 0.05), but stratified analyses did not show significant effects in persons with Hcy ≤18 or >18 µmol/L (data not shown). In addition, negative binomial regression showed that the intervention had no significant effect on the number of falls per participant (OR: 1.0; 95 % CI 0.9–1.1). Similar results were observed in the PP analyses, and after adjustment for confounders (data not shown). The 2-year change in Hcy was not associated with falling (HR:1.0; 95 % CI 1.0–1.0).

## Discussion

Two-year daily supplementation of vitamin B12 and folic acid in hyperhomocysteinemic persons >65 years was not effective in reducing the age-related decline in physical performance and handgrip strength. In addition, the intervention had no effect on the risk of falling. Despite the overall null-effect, the results provide indications for a positive effect of the intervention on walking as component of physical performance, as well as on physical performance among compliant persons >80 years.

This is the first study performed in a large sample of elderly, with moderate long-term follow-up duration. Although a higher decrease in Hcy was associated with higher follow-up performance scores, this effect was rather small and cannot be considered clinically relevant. The overall results are in line with previous findings of Lewerin et al. [19]. In that study, movement performance as indicated by a postural-locomotor-manual test did not improve after 4 months of treatment with 0.5 mg vitamin B12, 0.8 mg folic acid, and 3 mg vitamin B6 versus placebo in elderly participants (*n* = 209, mean age: 76 years (range 70–93 years), mean Hcy: 17.2 µmol/L). However, interaction effects with age were not examined in that study. In the randomized controlled trial of Sato et al., no differences in fall incidence between the intervention and placebo group were reported after 2-year supplementation of 1.5 mg vitamin B12 and 5 mg folic acid among a highly selective sample of stroke survivors (*n* = 628, mean Hcy: 19.9 µmol/L) [20]. Those findings are consistent with our results too.

Secondary analyses indicated that the intervention might have a positive effect on walking performance, in contrast to chair stands and tandem stand. The walking test includes aspects of coordination, proximal muscle strength, and balance, whereas the chair stands and the tandem stands only include aspect of proximal muscle strength and balance, respectively. Previously, it has been shown in observational data that high Hcy levels are most strongly associated with the walking test and the chair stands [15]. The current findings are partly in line with those previous observations.

In a subgroup of persons >80 years who were compliant in taking the supplement, a tendency toward a preventive effect on decline in physical performance was observed. The number of persons per treatment group in this analysis was relatively small (162 in the intervention and 178 in the placebo group). Although the results were not statistically significant, they might be clinically relevant. Previous studies showed that a change in the Short Physical Performance Battery (SPPB) (which also consists of a walking, chair stands and balance test) of 0.3–0.8 points may be considered as minimally clinically relevant among the elderly, and a change of 0.4–1.5 points as substantially clinically relevant [26, 27]. The currently used performance tests use different components than the SPPB, i.e., 6 m maximal walking speed vs 4 m habitual speed, and a different scoring of the balance test. However, it is similar with respect to the scorings range (0–12), and therefore the identified relevant change might be helpful in providing some indication of what can be considered as relevant change in our study. We believe that our observed change of 0.6 points might be even more relevant for persons >80 years as compared to persons ≤80 years, since baseline physical performance scores in this subgroup were significantly lower as compared to persons ≤80 years. Because the interaction effect with age was only observed in secondary analyses on a PP base, the subgroup effect on physical performance can be considered as a hypothesis that needs to be tested in further studies.

Recently, we have shown that B-vitamin supplementation was effective in the prevention of fractures in compliant persons >80 years [24]. This is in line with the observed borderline effect on physical performance in compliant persons >80 years in the current paper. The mechanism of action by which supplementation reduces the fracture risk remains uncertain. It has been suggested that bone mineral density, collagen cross-linking, osteoclast activity, and/or methylation capacity may be involved [28]. An alternative or complementary intermediate might be physical function. To test this hypothesis, we added physical performance scores to the fracture analyses in a post hoc analysis. In the compliant 80+ subgroup with data on physical performance, the HR for fractures in the intervention group was 0.3 and did not change after adjustment for physical performance scores. This indicates that physical performance cannot be considered as a mediator in the reported effect on fractures.

With respect to handgrip strength, we observed a decrease of 1.3 kg in the intervention group whereas in the placebo group a decrease of 1.4 kg was observed. A recent study with data from four intervention trials aimed at improving strength and physical function among postmenopausal women demonstrated that those with handgrip weakness at baseline had a better response to the interventions [29]. Such an interaction effect was not observed for lower extremity strength, indicating that the upper body may be more adaptable to interventions in older adults. Although handgrip strength is correlated with lower extremity strength or overall strength, it cannot replace the assessment of lower extremity strength [30]. In our study, indications for a greater treatment effect among those with handgrip weakness were not found, as the interaction of treatment effect with baseline handgrip strength was not significant.

Both the intervention and placebo tablets contained 600 IU vitamin D. In a meta-analysis of 13 studies, positive effects of vitamin D supplementation on muscle function have been demonstrated among elderly persons [31]. Another meta-analysis observed a reduced fall risk and rate of falls with vitamin D supplementation among the elderly [32]. Vitamin D receptors have been located in many tissues, such as muscle tissue and brain tissue [33, 34]. Age-related decline in vitamin D concentration and reduction of activity and expression of vitamin D receptors have been associated with reduced muscle cell function and are suggested to affect neuromuscular control and coordination [35, 36]. The presence of vitamin D supplementation in both treatment arms has most likely reduced the contrast between the groups and may have attenuated the results, although vitamin D and vitamin B12/folic acid supplementation are assumed not to have similar mechanistic actions.

Another limitation might be that the participants in the current study were only mildly hyperhomocysteinemic, with a few vitamin B12 (4 %) or folate (3 %) deficient participants, which may have diminished the effect of supplementation. In addition, the results might have been attenuated by the inclusion of participants that already used vitamin B12 and/or folic acid supplements (16 %), although we did not observe an interaction effect of B12 and/or folic acid supplement use. While we only observed an interaction effect of baseline Hcy with treatment for the outcome falling, with no significant effects in subsequent stratified analyses, the results might be different in case of severe hyperhomocysteinemia. In general, because the participants were screened for Hcy levels and Hcy levels have been linked to several adverse health outcomes (cardiovascular disease, cognitive decline, osteoporosis), the B-PROOF sample might be less healthy than the general population of older persons. On the other hand, persons who are willing to participate in nutrition-related randomized controlled trials are in general more healthy. The strength of this study is its randomized controlled study design, as well as the large number of included participants. In addition, the compliance to the treatment was good; the randomization resulted in two very well balanced groups; and the applied doses of vitamin B12 and folic acid were sufficient to lower Hcy levels [24]. It should be noted that more cancer diagnoses were observed in the intervention group as compared to placebo, as reported previously [24].

In conclusion, this study showed no overall effect of daily 2-year supplementation of vitamin B12 and folic acid on physical performance, handgrip strength, and falling among elderly persons with mild hyperhomocysteinemia. A tendency toward a positive effect on physical performance among compliant persons >80 years, as well as on gait was observed. As the loss of mobility in older persons forms an increasing public health issue in the context of rapidly expanding older populations, the current findings are important for advancing future research on preserving mobility in older persons. The current results emphasize the need for trials among the oldest old and on lower extremity functioning.
